# Entomopathogenic Fungi as Endophytes for Biological Control of Subterranean Termite Pests Attacking Cocoa Seedlings

**DOI:** 10.3390/jof6030126

**Published:** 2020-08-05

**Authors:** Chaba F. Ambele, Sunday Ekesi, Hervé D. B. Bisseleua, Olubukola O. Babalola, Fathiya M. Khamis, Christian T. L. Djuideu, Komivi S. Akutse

**Affiliations:** 1 International Centre of Insect Physiology and Ecology (icipe), P.O. Box 30772-00100 Nairobi, Kenya; felicitefavour@yahoo.com (C.F.A.); sekesi@icipe.org (S.E.); fkhamis@icipe.org (F.M.K.); 2 Food Security and Safety, Faculty of Agriculture, Science and Technology, North-West University, South Africa, Private Bag X2046, Mmabatho 2745, South Africa; olubukola.babalola@nwu.ac.za; 3 World Cocoa Foundation, P.O. Box 217 Accra, Ghana; herve.bisseleua@worldcocoa.org; 4 Zoology Laboratory, Faculty of Science, University of Yaoundé I, P.O. Box 812 Yaoundé, Cameroon; djuideuchristian@gmail.com

**Keywords:** colonization, fungal endophytes, inoculation techniques, relative pathogenicity, subterranean termite pests, virulence

## Abstract

This study was conducted in the scope of developing a sustainable effective approach against subterranean termite pests using entomopathogenic and endophytic fungus-based biopesticides. Termites, *Odontotermes* spp. workers, were tested for their susceptibility to 15 entomopathogenic fungal isolates through the direct spraying of conidia suspensions at 1 × 10^8^ conidia/mL. In general, all the isolates screened were pathogenic, with 100% mortality 4–7 days post-inoculation. However, the most virulent isolates were *Metarhizium brunneum* Cb15-III; the *M. anisopliae* isolates ICIPE 30 and ICIPE 60; *Hypocrea lixii* F3ST1; and the *Beauveria bassiana* isolates ICIPE 279, ICIPE 706 and ICIPE 662. These isolates were further tested for their endophytic colonization of cocoa seedlings using seed soaking, soil drench and foliar spray at 1 × 10^8^ conidia/mL. The colonization of the plant tissues by the fungi was determined using a culture-based technique. Only the *B. bassiana* isolates ICIPE 706 and ICIPE 279, and *H. lixii* F3ST1 colonized the cocoa seedlings, with varied colonization rates among isolates and inoculation methods. Three naturally occurring endophytes—*Trichoderma asperellum*, *Fusarium solani* and *F. redolens*—were also isolated from the cocoa seedling tissues. These findings suggest that cocoa seedlings are conducive to endophytic fungal growth either occurring naturally or from artificial inoculation Our findings could possibly lead to an innovative approach to the management of herbivory and subterranean termite pests in cocoa agroforests.

## 1. Introduction

Termites are becoming major pests in cocoa agroforests, causing significant losses [1,2,3,4]. Their control in the past relied on persistent organochlorine insecticides [5], which are now under restrictive use due to increasing concerns over negative effects on human and environmental health. Farmers in many parts of Africa use various traditional termite control methods, often with limited success [2]. The majority of termite control practices are ineffective and ecologically unsustainable and, above all, do not address the root attacks of termite infestation [2]. There is, therefore, a need for the identification of sustainable eco-friendly and locally available alternative methods for termite control in cocoa agroforests. Biological control using naturally occurring microorganisms such as entomopathogenic fungi (EPF) as part of integrated pest management could be the most sustainable long-term solution for termite control in cocoa agroforests. Entomopathogenic fungi (EPF) have long been considered as a promising approach for sustainable termite control [6]. This is because termites are assumed to live in environments conducive to entomopathogens [7,8]. The use of entomopathogens for termite control started as early as 1965 [9,10]. Most of these studies focused on the use of the EPF *Beauveria bassiana* (Balsamo) Vuillemin and *Metarhizium anisopliae* (Metschnikoff) Sorokin [6,8,11]. These EPF have generally been proved to be effective against termites in laboratory studies but have had little success in field trials when applied as conidial suspensions or granules [12]. This is due to repellency or host avoidance and because termites can deploy a diversity of defense mechanisms against the pathogens [13,14]. The efficacy of EPF against termite pests is also limited by abiotic factors (e.g., UV radiation, temperature and low humidity) that reduce the viability of fungal conidia [15]. Because of these reasons, the biological control of termites using EPF has generally been regarded as unfeasible [11,12,16]. 

However, apart from their direct mode of action against insect pests, several fungal entomopathogens have been reported to be able to grow internally within plant tissues, paving the way for possible use as endophytes in plant protection measures [17]. Mburu et al. [14] showed a co-evolutionary phenomenon in which termites’ behavioral response to either *M. anisopliae* or *B. bassiana* isolates is directly related to the potential infections that these fungi can inflict on them, and thus, termites can detect a virulent isolate from a distance and avoid direct contact. The foragers of the termite species *Coptotermes formosanus* Shiraki were significantly repelled by tree-based organic mulches coated with *M. anisopliae*, and the mulch consumption was highly reduced by up to 71% [18]. The repellent action of conidia against termites has also been utilized to protect maize crops from termites, which significantly reduced maize plant logging and increased grain yield in Kenya [19]. Therefore, another possible approach against subterranean termite pests of cocoa could be the inoculation of cocoa plants with highly virulent fungal entomopathogen as endophytes [20]. Several species of fungal entomopathogens are now known to have complex relationships with host plants as endophytes [15]. *Beauveria bassiana* for example, has been reported as an endophyte of maize [20,21], bananas [22], tomatoes [23], *Theobroma gileri* [24], coffee [25], and cocoa seedlings [26]. One advantage of the application of endophytes over the conventional direct application of fungal entomopathogens is the ability to colonize plants systemically, thereby offering continuous protection against insect pests. There is a plethora of recent studies on the insect-repellent, antifeedant and toxicity attributes of endophytic EPF [27,28,29]. However, whether virulent EPF against termites could colonize host tissues in cocoa seedlings and protect the seedlings against subterranean termites’ damage has not been evaluated. 

In the scope of investigating the potential use of EPF to control subterranean termite pests in cocoa agroforests, this study was designed to (1) screen and select virulent entomopathogenic fungal strains on key subterranean termite species attacking cocoa and (2) determine if the most virulent entomopathogenic fungal isolates could be established as endophytes in cocoa seedlings, thereby establishing an important first step in the possible use of virulent entomopathogenic fungal endophytes as biocontrol agents against termites in cocoa agroforests.

## 2. Materials and Methods

### 2.1. Termite Collection and Maintenance

Termites (*Odontotermes* spp.) were collected from infested wood around *icipe* Duduville campus, Nairobi, Kenya (1.221 S, 36.896 E; 1616 m above sea level) and placed on sterilized soil in plastic containers. They were then transferred into an incubator (26 ± 2 °C and 75 ± 5% RH in the dark) and kept for 1 h for acclimatization before being used in the bioassays. The relative humidity in the incubator was controlled using a saturated solution of K_2_SO_4_ (Supelco, Sigma-Aldrich, UK). Active and non-injured worker castes with a uniform size were selected for the various treatments.

### 2.2. Fungal Culture and Suspension Preparation

The identity, origin and source of the fungal isolates used in this study are summarized in Table 1. Six *Metarhizium anisopliae* fungal isolates, seven *Beauveria bassiana* isolates and one *Hypocrea lixii* isolate were obtained from the *icipe*’s Arthropod Pathology Unit Germplasm, while *M. brunneum* Cb15-III was obtained from Bielefeld University, Germany. The *Metarhizium* isolates were cultured on Sabouraud Dextrose Agar (SDA), while the *Beauveria* and *Hypocrea* isolates were cultured on Potatoes Dextrose Agar (PDA) medium in 90 mm Petri dishes in complete darkness at 25 ± 1 °C. Prior to bioassays, conidia were harvested from 2- to 3-week-old fungal cultures by scraping the plate surfaces with a sterile spatula. After harvesting, the conidia were suspended in 10 mL of sterile distilled water containing 0.05% Triton X-100 (Fluka, Sigma-Aldrich, UK) in 30 mL universal bottles. Five glass beads (ɸ = 3 mm) were added to each bottle to facilitate the breakdown of the hyphae. The bottles were stoppered and then vortexed in a Vortex (Genie 2 Scientific Industries, Bohemia, NY, USA) for 5 min at about 700 rpm to ensure a homogeneous conidial suspension. We then filtered the suspensions using cheesecloth to separate the conidia from the hyphae and to remove conidial clumps and mycelia debris. Conidial concentrations were quantified using an Improved Neubauer haemocytometer (Weder Scientific International Ltd., Teddington, UK) under a light microscope (Leica Microsystems, Cambridge, UK). The conidial suspensions were adjusted to 1 × 10^8^ conidia/mL through dilution with distilled water prior to the bioassays.

For the viability test, a concentration of 3 × 10^6^ conidia/mL was prepared, and 0.1 mL of the suspension was evenly spread on SDA or PDA plates. Four sterile microscope cover slips were randomly placed on the surface of each inoculated plate. The inoculated plates were then sealed with Parafilm and incubated at 25 ± 2 °C in complete darkness for 18 h. To stop the germination and assess the viability of the conidia, lactophenol cotton blue was added to the plates to stain the spores for ease of counting, following the method described by Goettel and Inglis [30]. One hundred (100) conidia were randomly counted under the cover slips at 400× magnification using a light microscope (Leica Microsystems, Cambridge, UK). Conidia were considered to have germinated when the length of the germ tube was at least twice the diameter of the conidium [30,31]. Four replicate plates were used per isolate, and viability of each isolate was determined, where more than 95% germination was obtained for all the isolates (Table 1).

### 2.3. Screening of Fungal Isolates for Time–Mortality Responses

Fifteen fungal isolates (7 *Metarhizium*, 7 *Beauveria* and 1 *Hypocrea*) were screened against the worker termites of *Odontotermes* spp. The termites used were larger workers involved in foraging, hence the most vulnerable to pathogens, and mostly act as carriers of pathogens [32]. Twenty (20) termites per treatment were placed in round plastic containers of 12 cm diameter × 6 cm. The control samples were sprayed with 10 mL of sterile distilled water containing 0.05% Triton X-100 (Fluka, Sigma Aldrich, UK) (without any fungal conidia) prior to fungal treatment inoculation using a Potter spray tower (Burkard Manufacturing Co Ltd., Rickmansworth, UK). For the fungal treatment groups, each plastic bowl containing the test termites was sprayed with 10 mL of a suspension of a concentration of 1 × 10^8^ conidia/mL. The spray tower was cleaned with 70% alcohol and sterile distilled water after each treatment. To maintain social cohesion within the group, two soldier termites were also added into each plastic bowl after conidial inoculation [33]. A piece of wet cotton wool was used to maintain high humidity in each plastic bowl throughout the experiment. The lids of the plastic bowls had aeration holes to ensure the free flow of air. Pieces of wood that the termites were found feeding on were collected, sterilized and added as food. Sterilized soil (autoclaved at 121 °C for 1 h), was provided as shelter after applications of conidia on the termites in each plastic bowl. The various treatment bowls were placed on moist cotton wool spread on a metal stand. The metal stand was immersed in a saturated solution of K_2_SO_4_ to maintain moisture in bigger plastic containers (34 × 24 × 20 cm). The plastic containers were then placed in an incubator maintained at 23 ± 2° and 75 ± 5% RH. Mortality was recorded daily for seven days, and the LT_50_ values (time needed to cause 50% mortality in the exposed termite population) for each of the fungal isolates and controls were calculated. 

To confirm that mortality was due to fungal infection, the cadavers were removed from treatments then surface-sterilized in 1% sodium hypochlorite solution and then in 70% alcohol for 3 s in each solution and rinsed for 3 min in sterile distilled water. They were then placed into 90 mm Petri dishes lined with Whatman filter paper moistened with sterile distilled water. The Petri dishes were covered with their lids, the edges sealed with Parafilm, and placed in an incubator. Mycosis was confirmed by daily microscopic examinations of hyphae and spores at a magnification of 400×. The cadavers were monitored for fungal growth for at least two weeks.

### 2.4. Cocoa Seeds and Seedlings

Cocoa seeds from ripe cocoa pods were obtained from the Institute of Agricultural Research for Development (IRAD), Yaoundé (Cameroon), and used for the inoculation experiments. All experiments were conducted at the International Centre of Insect Physiology and Ecology (*icipe*), Nairobi, Kenya. The cocoa seeds were surface sterilized by submersion in 70% ethanol for 2 min, followed by submersion in 1.5% sodium hypochlorite for 3 min, and rinsed in sterile distilled water thrice. The seeds were air-dried on sterile paper towels under a safe hood guard prior to inoculation or planting. Seedlings were grown on sterilized planting soil substrate mixed with livestock manure in a 5:1 ratio and at the density of one plant per pot.

### 2.5. Inoculation Methods

For seed inoculation, the sterilized cocoa seeds were soaked for 12 h in a conidial suspension of 1 × 10^8^ conidia/mL prepared from the most virulent isolates (*M. brunneum* Cb15-III; *Hypocrea lixii* F3ST1; *M. anisopliae* ICIPE 30 and ICIPE 60; and *B. bassiana* ICIPE 279 and ICIPE 706), obtained from the above pathogenicity bioassay. The control seeds were soaked in a sterile distilled water solution of 0.05% Triton X-100. The planting soil substrate was mixed with livestock manure in a 5:1 ratio, sterilized in an autoclave for 2 h at 121 °C, and allowed to cool down to ambient temperature for about 72 h before use. After soaking, each seedling was transferred individually into pots containing the sterile mixture of soil and manure in a screenhouse. The pots were watered thrice a week. The experiment with each isolate was replicated 4 times, and all the treatments were randomized in a complete block design.

For seedling inoculation, surface-sterilized seeds, as described above, were raised in plastic buckets with sterile planting substrate and maintained in a screenhouse at room temperature (25 ± 2 °C and 60% RH) for two months. Two inoculation methods (foliar spray and soil drench) were applied. For the soil drench method, each cocoa seedling root area was drenched with 25 mL of the fungal isolates’ inoculum mentioned above of each conidia suspension at the concentration of 1 × 10^8^ conidia/mL, applied to the soil surface around the base of the plants. Control seedlings were inoculated with 25 mL of sterile distilled water containing 0.1% Triton X-100. Each treatment was replicated four times, and the plants were maintained under conditions similar to those for seed inoculation. For foliar spray, a spore stock suspension of each isolate was prepared, and the spore concentration was adjusted to 1 × 10^8^ conidia/mL. The foliar application was performed with a hand sprayer using an average of 25 mL per plant. The spray was directed mainly to the leaves. The top of each pot was covered with aluminum foil while spraying to avoid conidial runoff to the soil. The experiment was arranged in a completely randomized design where the treatments and controls were replicated 4 times. After inoculation, the plastic containers containing the inoculated cocoa seedlings were kept in the screenhouse and watered thrice a week. 

### 2.6. Assessment of Colonization

To determine the colonization rates for each fungus-inoculated plant, evaluation was conducted at two months after inoculation, where four separate plants per isolate were used. The pots containing cocoa seedlings were transferred from the screenhouse to the laboratory, where the plants were uprooted and individually washed with running tap water. After washing, the plants were placed on paper towels on a clean bench to dry. Leaves were randomly selected and cut into small pieces (approximately 5 × 5 mm) using a sterile scalpel and forceps. Similarly, the stems and roots were also cut into small pieces (approximately 5 mm long). Five parts of each plant tissue were randomly selected, surface-sterilized in 70% ethanol for 2 min and 1.5% sodium hypochlorite suspension for 3 min, and rinsed three times in sterile distilled water. The surface-sterilized plant parts were placed on sterile filter paper on a clean working surface in a cabinet until the residual water had evaporated and then placed in a uniform distribution on SDA and PDA plates amended with antibiotics (tetracycline and streptomycin sulfate salt at 0.05%). The final rinse water was plated on SDA and PDA plates amended with antibiotics, and a tissue imprint was conducted to assess the effectiveness of the surface sterilization procedure [17]. The plates were evaluated for seven days after incubation. Plates with plant tissues were incubated at 25 °C, and the assessment of fungal endophyte occurrence was performed daily after the first day of incubation until 14 days after incubation. Positive colonization was scored by counting the number of pieces of the different plant parts with the growth of the inoculated endophytes. The inoculated fungal isolate, together with other fungal endophytes found, was isolated. The isolated fungi were sub-cultured five times to obtain pure cultures. To confirm whether the growing endophytes were the ones initially inoculated, slides were prepared from the mother and re-isolated sub-cultured plates used for morphological comparison [34,35]. The isolated fungal endophytes that occurred naturally from the plant tissues were identified using molecular tools. 

### 2.7. Morphological Identification of the Inoculated Endophytes

The fungal endophytes that were isolated from the cocoa seedling tissues were sub-cultured five times to obtain pure cultures. Morphological identification was performed following procedures described by Akutse et al. [36], Goettel and Inglis [30], Humber [34,35], and Burgess et al. [37]. These procedures were used to assess the major fungal colony features (appearance, texture and pigmentation) by examining the top and reverse of the plates. In addition, slides were also prepared from the pure cultures and used for observing key fungal characteristics such as mycelium type, microconidia and macroconidia, conidium size and shape, and chlamydospore and microconidia chains. 

### 2.8. Molecular Identification of Naturally Occurring Endophytes Isolated from Cocoa Seedlings

#### 2.8.1. DNA Extraction and PCR Amplification

For the molecular identification of the three isolated fungal endophytes from cocoa seedling tissues, the fungal colonies were harvested from two-week-old cultures by scrapping the conidia into 1.5 mL Eppendorf tubes containing three sterile beads. We extracted the Genomic DNA using a Plant DNA extraction kit (Bioline, London, UK) following the manufacturer’s instructions. The extracted DNA was quantified using a Nanodrop 2000/2000c spectrophotometer (Thermo Fischer Scientific, Wilmington, USA). The samples were then stored at −20 °C until further processing.

The universal fungal ITS primers—forward primer ITS5, (5′-TCCTCCGCTTATTGATATGC-3′), and reverse primer ITS4, (5′-GGAAGTAAAAGTCGTAACAAGG-3′) [38]—were used to carry out the PCR amplification of the fungal isolates. A final volume of 20 µL containing 5× MyTaq reaction buffer (Bioline, London, UK) (5 mM dNTPs, 15 mM MgCl2, stabilizers and enhancers), 0.5 pmol µL^−1^ of each primer, 0.25 mM MgCl2, 0.0625 U µL^−1^ of MyTaq DNA polymerase (Bioline, London, UK), and 15 ng µl^-1^ of DNA template was used to carry out all the PCR reactions [36]. A Mastercycler Nexus thermal cycler (Eppendorf, Hamburg, Germany) was used to set up the PCR reactions, with the cycling conditions of (i) an initial denaturation step at 95 °C for 10 min, (ii) 35 cycles of a denaturation step at 94 °C for 1 min, and (iii) an annealing step at 54 °C for 1 min, followed by (iv) an extension step at 72 °C for 1 min and (v) an extension at 72 °C for 10 min. The expected product size ranged between 450 and 600 bp [36]. 

A 1.2% agarose gel was used to resolve the amplified PCR products at 70 V for 80 min. Using a KETA GL imaging system trans-illuminator (Wealtec Corp, Meadowvale Way Sparks, Nevada, USA), the obtained DNA bands from the gel were analyzed and documented. Following the manufacturer’s instructions, an Isolate II PCR and Gel Kit (Bioline, London, UK) was used to excise and purify the successfully amplified products. 

#### 2.8.2. Sequencing and Analysis

The purified samples were shipped to Macrogen Inc Europe Laboratory, the Netherlands, for bi-directional sequencing. The sequences obtained were assembled and edited using the BioEdit Sequence Alignment Editor Version 7.2.5 software [39], and multiple alignment was performed in Clustal X (version 2.1) [40]. For conclusive identification, the sequences were queried via BLAST at the GenBank database hosted by NCBI [41,42,43].

### 2.9. Data Analysis

Mortality data were corrected for natural mortality in controls using Abbott’s formula [44]. Time-mortality data were analyzed with the Generalized Linear Model (GLM), using the function “dose.p” from the MASS library, to estimate the lethal time to 50% mortality (LT_50_) for each isolate that caused more than 50% mortality. The corrected percent mortalities of the worker termites 3 days after inoculation were calculated using a binomial generalized linear model with logit link [45]. Mycosis data were also analyzed using one-way analysis of variance (ANOVA). Prior to performing ANOVA, the assumption of homogeneity of variance was tested and satisfied using Bartlett’s test. 

The success rate (%) of the fungal colonization of the host plants’ parts was calculated following the Fisher and Petrini [46] formula:

$$\mathrm{Colonization}\;\%=\frac{\mathrm{Number}\;\mathrm{of}\;\mathrm{pieces}\;\mathrm{exhbiting}\;\mathrm{fungal}\;\mathrm{growth}}{\mathrm{Total}\;\mathrm{number}\;\mathrm{of}\;\mathrm{pieces}\;\mathrm{plated}\;\mathrm{out}}\;\times 100$$

The percentage values of plant colonization were square-root transformed to stabilize the variance, and the transformed data were then subjected to ANOVA. When there were significant differences between treatments, the means were separated using Student–Newman–Keuls (SNK) post hoc tests. All analyses were performed using R version 3.6.1 [47].

## 3. Results

### 3.1. Pathogenicity of EPF Isolates against Termites

**Figure 1:**
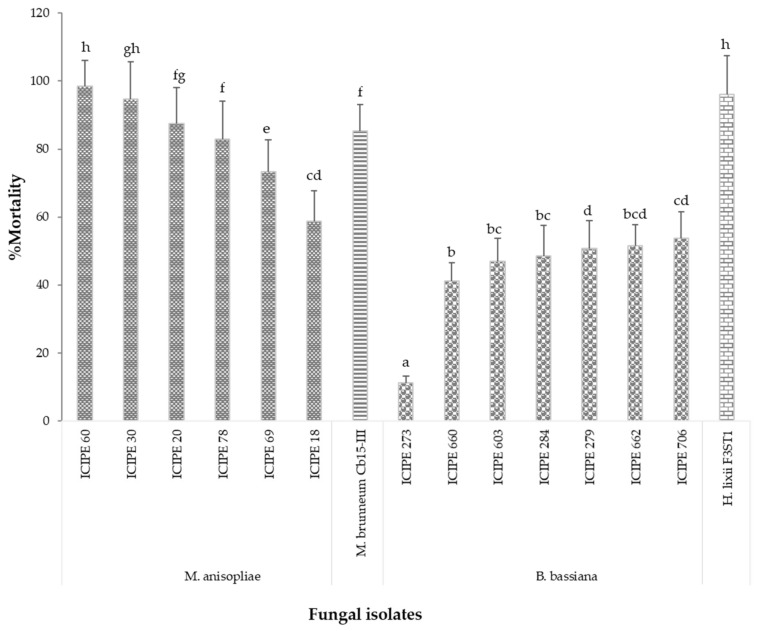
Mean percent mortality after 3 days post-inoculation of worker caste of *Odontotermes* spp. with fungal isolates of *Metarhizium anisopliae*, *M. brunneum*, *Beauveria bassiana* and *Hypocrea lixii.* Bars with different letters are significantly different (Tukey’s HSD, *P* ˂ 0.05).

The screening for pathogenicity of the 15 isolates at the concentration of 1 × 10^8^ conidia/mL indicated that all the isolates were pathogenic to the termites, causing a significantly higher mortality level (100%) between 4 and 7 days. The calculated LT_50_ values for the isolates (Table 2) and the corrected percent mortality of the worker termites three days after inoculation (Figure 1) showed that, in general, the *Metarhizium* isolates elicited quicker mortality with lower LT_50_ (1.50–2.84 days) values than the *Beauveria* isolates (2.23–4.41 days). 

The most virulent isolates with LT_50_ less than two days were *M. brunneum* Cb15-III, *M. anisopliae* isolates (ICIPE 60 and ICIPE 30) and *Hypocrea lixii* F3ST1, followed by isolates with LT_50_ between 2 and 3 days, and these included *M. anisopliae* isolates (ICIPE 20, ICIPE 18, ICIPE 78 and ICIPE 69) and *B. bassiana* isolates (ICIPE 279, ICIPE 284, ICIPE 706, ICIPE 662 and ICIPE 660), while *B. bassiana* isolates (ICIPE 603 and ICIPE 273) were the least virulent with LT_50_ of more than 3 days (Table 2). There were significant differences (F = _18.38_, df = 14, *P* = 0.001) in the mycoses of cadavers among the isolates (Table 2).

Generally, the sign of penetration of conidia observed on the termite cuticle treated with *Metarhizium* isolates appeared faster than on those treated with *Beauveria* and *Hypocrea* isolates. After the penetration, the conidia emerged on the surfaces of the cadavers. Termites started to change in colour after few days of infection (became darker) and became more fragile when touched.

### 3.2. Endophytic Colonization of Cocoa Seedlings by Artificially Inoculated Fungal Isolates

**Figure 2:**
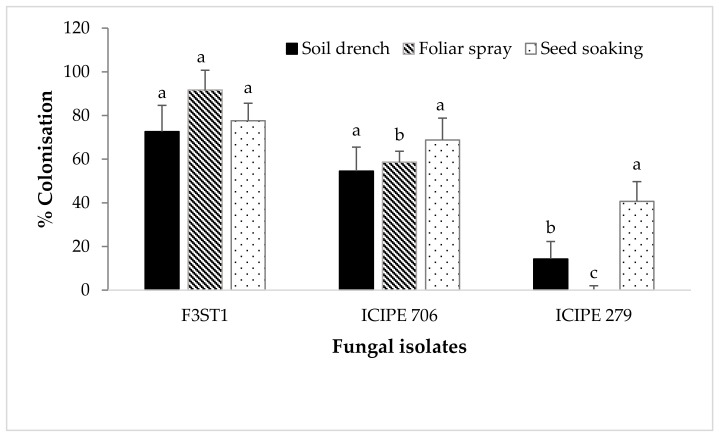
Mean (±SE) percentage of colonization of cocoa seedlings by different fungal pathogens (*Beauveria bassiana* ICIPE 279 and 706 and *Hypocrea lixii* F3ST1), 2 months post-inoculation using different inoculation methods (soil drench, seed soaking and foliar spray). Bars with different letters differ significantly at *P* = 0.05 (SNK test after ANOVA).

For the endophytic colonization experiment, all the *Metarhizium* isolates tested did not colonize any of the cocoa seedling parts. However, only the *B. bassiana* isolates (ICIPE 706 and ICIPE 279) and *H. lixii* F3ST1 were able to successfully colonize the cocoa seedlings. The last rinse water used to rinse the tissues after sterilization, the imprinting plants and the sterilized soil yielded no microorganisms, thus confirming that the external surface of the plant parts was properly surface sterilized and that any ensuing fungal growth from surface-sterilized tissues was inferred to have originated from internal plant tissues (i.e., as endophytes). Significant variations were observed in the percentage colonization between the different fungal isolates with regard to the inoculation method used (Figure 2). For the seed soaking method, no significant differences (F_2,17_ = 3.58, *P* = 0.05) were observed in the colonization of the different plant parts by the various fungal isolates (Figure 2). However, significant differences were observed in the colonization of the different isolates when the cocoa seedlings were sprayed with the fungal inoculum (F_2,19_ = 4.9, *P* < 0.001), as well as with the soil drench method (F_2,33_ = 10.76, *P* = 0.0002) (Figure 2).

**Figure 3:**
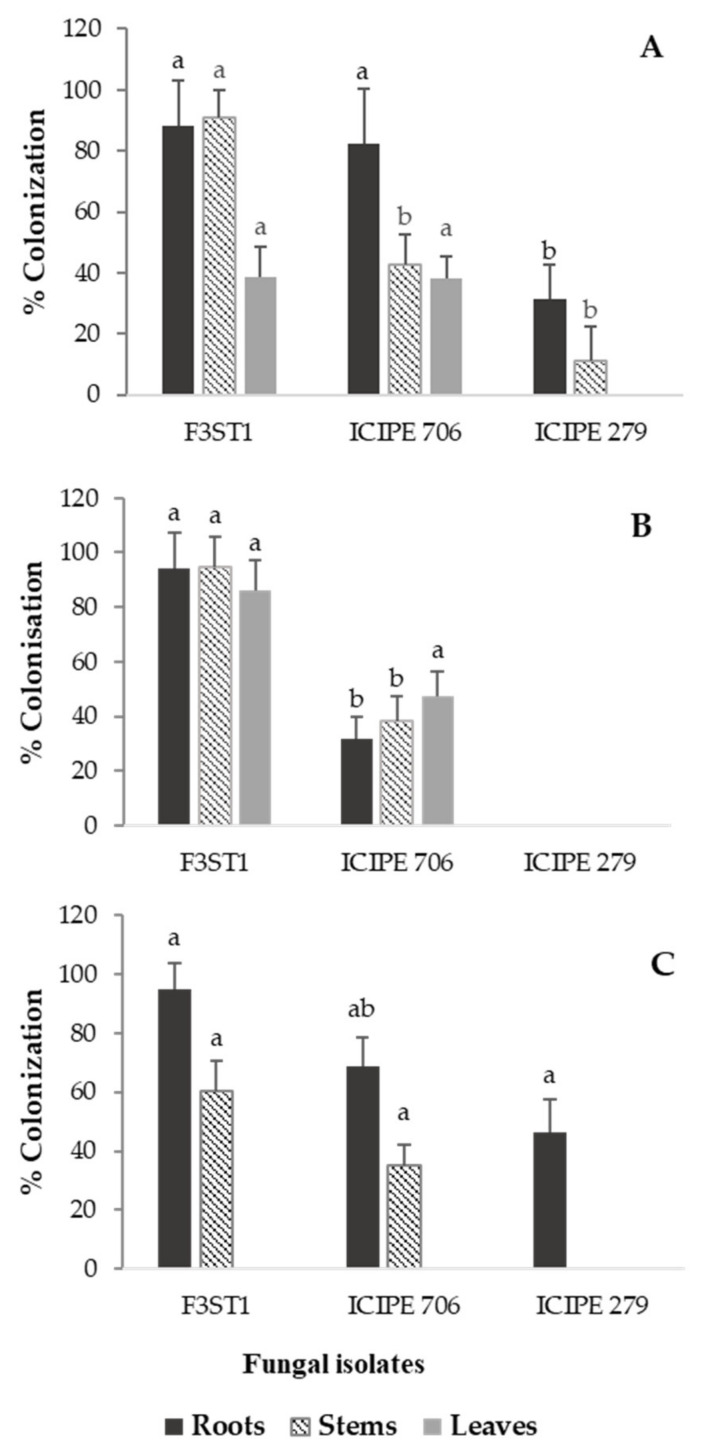
Mean (±SE) percentage of colonization of different cocoa seedling parts (root, stem and leaves) by fungal pathogens, *Beauveria bassiana* ICIPE 279 and 706 and *Hypocrea lixii* F3ST1, 2 months post-inoculation using different inoculation methods; (**A**) = soil drench, (**B**) = foliar spray and (**C**) = seed soaking. Bars with different letters differ significantly at *P* = 0.05 (SNK test after ANOVA).

The endophytic colonization of the cocoa seedlings also varied significantly between the fungal isolates with regard to the plant parts and the inoculation methods. The interaction between the percent colonization of the different plant parts and the method of inoculation of conidia was significant (F_4,97_ = 4.382, *P* = 0.003). For instance, for the soil drench method (Figure 3A), *H. lixii* F3ST1 showed the highest percent colonization for roots (88.2%) and stems (90.8%) but lower colonization for leaves (38.7%), while ICIPE 706 showed the highest colonization for roots (82.5%) but lower percent colonization for the leaf (38.2%) and stem (42.8%). ICIPE 279 showed no colonization for leaf parts but the lowest colonization for the stem (11.2%) and roots (31.6%). Significant differences (F_2,9_ = 6.18, *P* = 0.02) were observed for the root colonization of the different isolates in the soil drench method, where more root parts were colonized by *H. lixii* F3ST1 and ICIPE 706 than ICIPE 279. More stem parts (F_2,9_ =11.1, *P* = 0.003) were also colonized by *H. lixii* F3ST1 than ICIPE 706 and 279. However, no significant differences (F_2,9_ = 2.17, *P* = 0.17) were observed for the colonization of the leaf parts. For the foliar spray method (Figure 3B), *H. lixii* F3ST1 showed high colonization of the roots (94.3%), stems (94.7%) and leaves (82.2%). ICIPE 706 exhibited higher colonization of the leaves (47.4%) than stems (38.2%) and roots (31.6%), while no colonization was observed for any of the plant tissues for ICIPE 279. For this method, more root parts (F_2,9_ = 4.87, *P* = 0.0005) were colonized *by H. lixii* F3ST1, as well as the stem parts (F_2,9_ = 2.54, *P* < 0.001) and leaves (F_2,9_ = 12.67, *P* = 0.002), than ICIPE 706, whereas no plant part was colonized by ICIPE 279. For the seed soaking method (Figure 3C), *H. lixii* F3ST1 showed high colonization for roots (94.7%) and stems (60.5%), and ICIPE 706 colonized the roots (68.7%) and stems (35.2%), while ICIPE 279 colonized only the roots (46.2%). No isolate colonized the leaves in the seed soaking method of inoculation (Figure 3C). *Hypocrea lixii* F3ST1 significantly colonized the roots (F_2,19_ = 4.5, *P* = 0.004) and stem (F_2,9_ = 5.2, *P* = 0.03) compared to ICIPE 706 and ICIPE 279.

### 3.3. Naturally Occurring Fungal Endophytes Isolated from the Cocoa Seedlings

In addition to the artificially inoculated isolates that successfully colonized the cocoa seedlings (*B. bassiana* isolates ICIPE 279 and 706 and *H. lixii* F3ST1), three additional fungal endophytes that naturally occurred and were identified using molecular techniques as *Trichoderma asperellum*, *Fusarium redolens* and *F. solani* (Table 3), were isolated from the leaves, stems and roots. 

## 4. Discussion

All the fungal isolates screened were pathogenic to the termites, causing 100% mortality within 4–7 days post-inoculation. However, *M. brunneum* Cb15-III, *M. anisopliae* ICIPE 60 and ICIPE 30, and *Hypocrea lixii* F3ST1 were able to kill 50% of the exposed population in less than two days, showing high virulence to *Odontotermes* spp. The results obtained in this study corroborate those of other studies, which showed that many fungal isolates are virulent to termites [8]. For instance, Zoberi and Grace [48] showed that 100% mortality of *Reticulitermes flavipes* Kollar (Isoptera: Rhinotermitidae) workers was attained at 1–3 days after exposure to a strain of *B. bassiana* isolated from *R. flavipes* workers. Similarly, under laboratory conditions, a strain of *M. anisopliae* has been reported to cause 100% mortality to *Odontotermes formosanus* Shiraki (Isoptera: Termitidae) three days post-inoculation at the concentration of 3 × 10^8^ conidia/mL [49]. Hoe et al. [50] reported that isolates of *M. anisopliae* were pathogenic against a subterranean termite, *Coptotermes curvignathus*, causing 100% mortality at 1 × 10^7^ conidia/mL within three days post-inoculation. Kramm and West [7] also reported that 100% mortality occurred within one day after the exposure of termites to *M. anisopliae*, while similar mortality rates were obtained within five days in the case of *B. bassiana*. Following this high susceptibility of termites to EPF, researchers suspect that the rapid killing by EPF such as *M. anisopliae* of their hosts could be caused not only through the direct physical invasion of the hyphae but also possibly due to some enzymatic mechanisms or toxic metabolites produced by the EPF [33]. In addition, the soft nature of the termite cuticle could also contribute a lot to the rapid killing by EPF.

Although all the isolates screened were pathogenic, there was significant variation in their virulence levels. Our results showed that *Metarhizium* and *Hypocrea* isolates elicited high mortality with quicker killing speed and lower LT_50_ values than *Beauveria* isolates. The higher virulence effect of *Metarhizium* isolates than that of *Beauveria* isolates confirms earlier reports on similar patterns of activity with these fungal pathogen species [7,13,51,52,53,54,55]. *Metarhizium brunneum* Cb15-III, *M. anisopliae* ICIPE 30 and ICIPE 60, and *H. lixii* F3ST1 were the most virulent against the tested termite species. A previous study has reported the virulence of *M. brunneum* Cb15-III encapsulated with CO_2_ for the control of subterranean termite pests [32]. Other studies have also reported that the *Metarhizium* genus, especially *M. anisopliae* and *M. brunneum*, is one of the EPF genera that has high potential for controlling subterranean termites [56,57]. Research conducted by Milner et al. [58] also revealed *M. anisopliae* to be the most effective fungal pathogen for termite control. However, highly virulent isolates may not be always ideal candidates for biological control programs, especially in the case of social insects such as termites, because other reports have shown that the conidia of highly virulent strains of EPF are very repellent to termites [14,59,60]. Therefore, it is very important to assess the systemic use of these virulent EPF through the artificial inoculation of the host plant to confer resistance against the subterranean termites.

Our results show that only *B. bassiana* isolates ICIPE 279 and 706, and *H. lixii* F3ST1 could successfully colonize and establish in cocoa seedlings as endophytes without causing any visual/visible symptoms or damage to the seedlings like in the control treatments. *Beauveria bassiana* has also been artificially introduced into many other plants such as the common bean [61,62], maize [21], cocoa [26], date palms [63], coffee [64], bananas [22], radiata pine [65], faba beans [66], opium poppies [67], cotton [68,69], tomatoes [69] and grapevine leaves [70]. Similarly, *H. lixii* F3ST1 has been shown to endophytically colonize the common bean [62,71], onion [72] and faba bean [71,73]. This suggests that these entomopathogens have the potential to also endophytically colonize several host plant species without causing any visual detrimental effects on the host plants. This observation corroborates with the findings of Posada and Vega [26], who also did not observe any visual symptoms on cocoa seedlings colonized by *B. bassiana* and, therefore, concluded that *B. bassiana* can colonize cocoa seedlings without causing any detriment to the hosts. Additionally, it is also reported that entomopathogenic fungi can colonize different plant tissues as symptomless endophytes [27]. None of the *Metarhizium* isolates colonized the cocoa seedlings in this study. Akutse et al. [71] and Mutune et al. [62] also reported a lack of colonization and establishment of *M. anisopliae* isolates in *V. faba* or *P. vulgaris* cultivars through seed inoculation. *Metarhizium* species are less well known as endophytes [74], even though some strains have been successfully introduced into tomatoes [75], fava beans [66], oilseed rape [76] and haricot beans [77,78]. Two unidentified *Metarhizium* species and *M. anisopliae* have also been found to act as endophytes by naturally colonizing the roots of wall barley (*Hordeum murinum* L.) [79].

Our soil drench inoculation method resulted mostly in the colonization of roots and stems compared to the leaves, while the foliar spray inoculation method resulted mostly in the colonization of the leaves and stems as compared to the roots. Some studies have shown that colonization by the applied EPF is more likely in the plant part that was in direct contact with the inoculum and less likely in plant parts distant from the application site [80,81]. The variation in the colonization rates among the tested fungal strains, as shown in this study, may be due to the differential growth rate and endophytic abilities of each fungal isolate as associated with different host plant species or cultivars [71,82]. For example, *M. brunneum* Cb15-III, which did not colonize the cocoa seedling in this study, had been shown to be an efficient endophyte in wheat (*Triticum aestivum* L.) [70] and faba beans [83].

Previous studies have reported the endophytic colonization of plants with *B. bassiana* for as long as eight months in coffee [25] and nine months in radiata pine [65]. It therefore remains to be tested whether the endophytic colonization of cocoa seedlings by *B. bassiana* isolates ICIPE 279 and 706, and *H. lixii* F3ST1 can persist for more than one month and confer protection of cocoa seedlings against termite damage in the field following transplantation. Plant colonization by *B. bassiana* has been reported to reduce the damage caused by many crop pests such as the lepidopteran cob and the stemborers *Ostrinia nubilalis* and *Sesamia calamistis* Hampson (Lepidoptera: Noctuidae) in maize [21,52]; the banana weevil, *Cosmopolites sordidus* Germar (Coleoptera: Curculionidae) in bananas [22]; the poppy stem gall wasp, *Iraella luteipes* Thompson (Hymenoptera: Cynipidae) in opium poppy [84]; and the stem weevil *Apion corchori* Marshall (Coleoptera: Curculionidae) in white jute [82]. Adverse effects against insect pests have also been reported as a result of plant colonization with other fungal endophytes, such as the black bean aphid, *Aphis fabae* Scopoli (Hemiptera: Aphididae), and the pea leafminer, *Liriomyza huidobrensis* Blanchard (Diptera: Agromyzidae), with *H. lixii* in broad beans [71,80]; the cotton aphid, *Aphis gossypii* Glover (Hemiptera: Aphididae), with *B. bassiana* or *Lecanicillium lecanii* in cotton [68]; onion thrips, *Thrips tabaci* Lindeman (Thysanoptera: Thripidae), with *Clonostachys rosea*, *H. lixii*, *Trichoderma harzianum*, *T. asperellum*, *T. atroviride* and *Fusarium* sp. in onions [85]; and the bean stem maggot, *Ophiomyia phaseoli* Tryon (Diptera: Agromyzidae), with *H. lixii*, *T. asperellum* and *T. atroviride*, in beans [62]. Future studies are therefore warranted to assess the fate of the *B. bassiana* isolates ICIPE 279 and 706 and *H. lixii* F3ST1, which successfully colonized the cocoa seedlings, and the ability of these endophytes to confer systemic resistance against termite pests from the laboratory to in the field.

*Trichoderma asperellum*, *Fusarium redolens* and *F. solani* were also isolated from the cocoa seedling parts as endophytes. *Trichoderma* species are typically considered soilborne organisms associated with many plant parts [86]. Other researchers have also isolated *Trichoderma* and *Fusarium* spp. from live sapwood immediately below the bark of trunks of wild and cultivated *Theobroma cacao* and other *Theobroma* species [24]. These fungi therefore appear to be cocoa-associated endophytes possibly transmitted through the seeds. The pathogenicity of these fungal endophytes isolated from cocoa seedlings for termites and other cocoa pests also remains important for investigation.

## 5. Conclusions

This study suggests that *M. brunneum* Cb15-III and *M. anisopliae* isolates ICIPE 60 and ICIPE 30 have the potential to be developed as microbial termiticides for controlling subterranean termite pests in cocoa agroforests. The recovery of *B. bassiana* isolates ICIPE 706 and 279 and *H. lixii* F3ST1 from cocoa tissues after artificial inoculation through seed soaking, soil drench and foliar spray indicates that these fungi could establish as cocoa endophytes when inoculated. In addition, the lack of any visual symptoms (retarded growth, discoloration, lesions, etc.) on the seedlings also indicate that fungal isolates can colonize cocoa plants without causing any detrimental effects on the plant. Future studies are warranted to determine the growth parameters of the endophytically colonized seedlings, the fate of these isolates in cocoa seedlings transplanted in the field and their ability to protect the plant against subterranean termite pest damage in cocoa plantations.

## Figures and Tables

**Table 1:** Identity and % germination of fungal isolates screened against the termite *Odontotermes* spp. for virulence under laboratory conditions.

Fungal Species	Isolates	Source	Locality/Country	Year of Isolation	% Germination
*Metarhizium anisopliae*	ICIPE 20	Soil	Migori (Kenya)	1989	98.25 ± 0.47 bcd
	ICIPE 30	*Busseola fusca*	Kendubay (Kenya)	1989	98.06 ± 0.47 cde
	ICIPE 60	Soil	Kakelo-Seme (Kenya)	1990	99.10 ± 0.29 abc
	ICIPE 18	Soil	Mbita (Kenya)	1989	99.77 ± 0.01 a
	ICIPE 69	Soil	Matete (DRC)	1990	99.34 ± 0.28 ab
	ICIPE 78	*Temnoschoita quadripustulata*	Ungoe (Kenya)	1990	99.09 ± 0.03 abc
*Metarhizium brunneum*	Cb15-III	-	Germany	-	95.25 ± 0.48 f
*Beauveria bassiana*					
	ICIPE 273	Soil	Mbita (Kenya)	2006	96.96 ± 0.27 e
	ICIPE 279	Coleopteran larva	Kericho (Kenya)	2005	98.25 ± 0.85 bcd
	ICIPE 284	Coleopteran larva	Mauritus	2005	99.22 ± 0.38 ab
	ICIPE 603	Hymenoptera	Taita (Kenya)	2007	97.26 ± 0.48 de
	ICIPE 706	Monocots	Kenya	2008	99.51 ± 0.01 a
	ICIPE 660	Soil	Chemokock (Kenya)	2008	98.70 ± 0.06 abc
	ICIPE 662	Soil	Mariakani (Kenya)	2008	99.33 ± 0.14 ab
				
*Hypocrea lixii*	F3ST1	Maize	Kenya	2009	95.25 ± 0.45 f

Means within a column followed by the same letter are not significantly different according to Student–Newman–Keuls (SKN) tests (*P* ˂ 0.05).

**Table 2:** Median lethal time (LT_50_) 7 days after treatment and % mycosis of worker termites of *Odontotermes* spp. inoculated with different isolates of entomopathogenic fungi.

Fungal Species	Isolates	LT_50_ (Days) (95% FL)	% Mycosis of Termite Cadavers
***Beauveria bassiana***	ICIPE 279	2.23 (2.19–2.27)	66.25 ± 5.91 a
	ICIPE 603	3.09 (3.04–3.14)	47.50 ± 5.20 e
	ICIPE 284	3.00 (2.96–3.04)	61.25 ± 9.44 ab
	ICIPE 706	2.85 (2.82–2.88)	61.25 ± 4.27 ab
	ICIPE 662	2.85 (2.82–2.88)	70.00 ± 4.56 a
	ICIPE 660	3.00 (2.96–3.04)	62.50 ± 3.23 ab
	ICIPE 273	4.41 (4.37–4.45)	46.25 ± 5.54 bcd
***Metarhizium anisopliae***	ICIPE 20	2.23 (2.19–2.27)	48.75 ± 9.66 de
	ICIPE 60	1.51 (1.49–1.53)	52.50 ± 7.77 abcd
	ICIPE 30	1.72 (1.69–1.75)	41.25 ± 3.15 cde
	ICIPE 18	2.84 (2.80–2.88)	53.75 ± 2.39 abcd
	ICIPE 78	2.38 (2.35–2.41)	47.50 ± 4.33 bcd
	ICIPE 69	2.41 (2.38–2.44)	67.50 ± 6.61 abc
***Metarhizium brunneum***	Cb15-III	1.50 (1.44–1.56)	57.50 ± 10.9 bcd
***Hypocrea lixii***	F3ST1	1.80 (1.51–2.09)	62.54 ±7.11 ab

Means within a column followed by the same letters are not significantly different according to Student–Newman–Keuls (SNK) tests (*P* < 0.05). LT_50_ (in days); FL represents fiducially limit at 95%.

**Table 3:** Identified fungal endophytes species using ITS 5 and 4.

Sample Code	Identified Fungal Samples from GenBank	Query Coverage %	E-value	Identities (%)	GenBank ID	Sample Accession Numbers
CU	*Trichoderma asperellum* isolate NBAIR TATP small subunit ribosomal RNA gene, partial sequence; internal transcribed spacer 1, 5.8S ribosomal RNA gene, and internal transcribed spacer 2, complete sequence; and large subunit ribosomal RNA gene, partial sequence	100	0	99.82	MN727373.1	MT767125
CL	*Fusarium solani* isolate 7184.01 internal transcribed spacer 1, partial sequence; 5.8S ribosomal RNA gene and internal transcribed spacer 2, complete sequence; and large subunit ribosomal RNA gene, partial sequence	99	0	100	MN729431.1	MT767126
CR1	*Fusarium redolens* strain T1ST190421511 small subunit ribosomal RNA gene, partial sequence; internal transcribed spacer 1, 5.8S ribosomal RNA gene, and internal transcribed spacer 2, complete sequence; and large subunit ribosomal RNA gene, partial sequence	100	0	99.64	MN486568.1	MT767127
